# The Participation of Older People in the Concept and Design Phases of Housing in The Netherlands: A Theoretical Overview

**DOI:** 10.3390/healthcare9030301

**Published:** 2021-03-09

**Authors:** Joost van Hoof, Katja M. Rusinovic, Zsuzsu. K. C. T. Tavy, Rudy F. M. van den Hoven, Jeroen Dikken, Suzan van der Pas, Hanneke Kruize, Simone R. de Bruin, Marianne E. van Bochove

**Affiliations:** 1 Faculty of Social Work & Education, The Hague University of Applied Sciences, Johanna Westerdijkplein 75, 2521 EN Den Haag, The Netherlands; z.k.c.t.tavy@hhs.nl (Z.K.C.T.T.); r.f.m.vandenhoven@hhs.nl (R.F.M.v.d.H.); j.dikken@hhs.nl (J.D.); 2 Faculty of Public Management, Law & Safety, The Hague University of Applied Sciences, JohannaWesterdijkplein 75, 2521 EN Den Haag, The Netherlands; k.m.rusinovic@hhs.nl (K.M.R.); vanbochove@eshpm.eur.nl (M.E.v.B.); 3 Faculty of Health, Nutrition & Sport, The Hague University of Applied Sciences, Johanna Westerdijkplein 75, 2521 EN Den Haag, The Netherlands; 4 Faculty of Social Work & Applied Psychology, University of Applied Sciences Leiden, Zernikedreef 11, 2333 CK Leiden, The Netherlands; pas.vd.s@hsleiden.nl; 5 Centre for Sustainability, Environment and Health, National Institute for Public Health and the Environment, Antonie van Leeuwenhoeklaan 9, 3721 MA Bilthoven, The Netherlands; Hanneke.kruize@rivm.nl; 6 Research Group Living Well with Dementia, Department of Health and Wellbeing, Windesheim University of Applied Sciences, Campus 2, 8017 CA Zwolle, The Netherlands; sr.de.bruin@windesheim.nl; 7 Erasmus School of Health Policy & Management (ESHPM), Burgemeester Oudlaan 50, 3000 DR Rotterdam, The Netherlands

**Keywords:** homes, older adults, engagement, age-friendly, ageing in place, methodology, governance

## Abstract

In the Netherlands, there is a growing need for collective housing for older people to bridge the gap between ageing-in-place and institutional care facilities. Participation of older people in the concept and design phases is important to tune the market supply to the needs of (future) residents, yet social entrepreneurs find it challenging to involve older people. This commentary explores various ways older people can participate in the development of new housing initiatives. The ladder of citizen participation is applied to explore different roles that (future) residents could play with levels of influence varying from non-participation to citizen power. Considerations for meaningful participation are discussed, in order to show how collaborations can be formed between (future) residents and decision makers.

## 1. Introduction

All over the Western World, people live longer and are generally in better health than previous generations of their age. According to the Organisation for Economic Co-operation and Development (OECD) [1], the population share of people aged 65 years and over is expected to rise to more than 25% in 2050 across its member states. Cities in particular have large numbers of older inhabitants and are home to about 45% of this older population. The interaction of ageing and urbanism, which is also termed urban ageing [2,3], raises issues for all types of communities in various domains of urban living [4,5,6,7,8,9]. An age-friendly city is a place where older people are actively involved, valued, and supported with infrastructure and services that effectively accommodate their needs [3].

**Figure 1:**
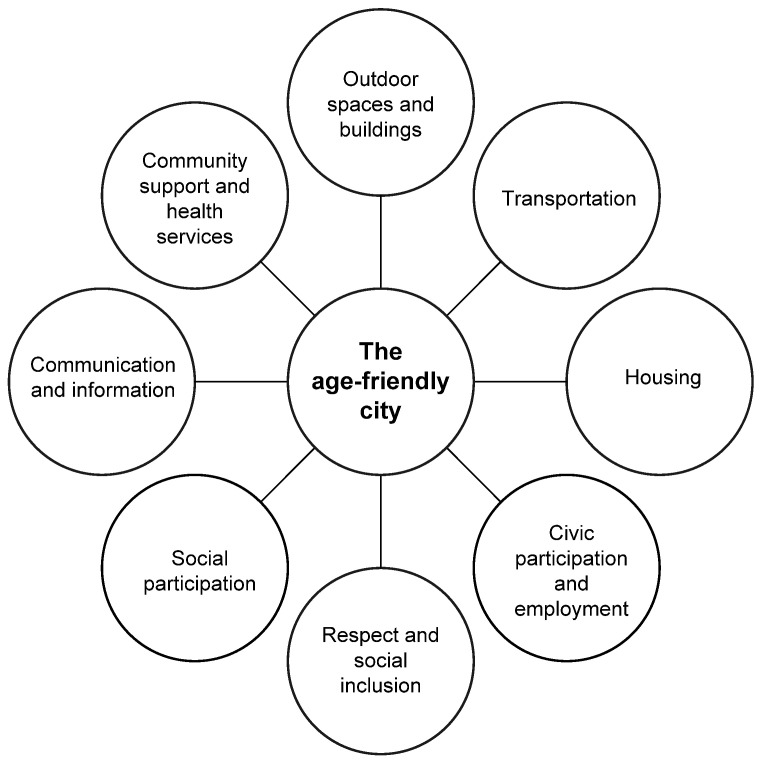
The eight domains of an age-friendly city [10].

The World Health Organization (WHO) engages and assists cities in becoming more ”age-friendly”, through the Global Age-Friendly Cities Guide and a companion ”Checklist of Essential Features of Age-Friendly Cities” [10]. There are eight domains of an age-friendly city (Figure 1). An ”age-friendly” city aims to optimise opportunities for health, participation, and security in order to enhance the quality of life of residents as they age. According to the OECD [1], ageing societies pose diverse challenges, such as redesigning infrastructure, transport and urban development patterns, social isolation, lack of accessibility and affordable housing.

Housing is one of the eight domains of the WHO’s model of age-friendly cities and communities. The establishment of appropriate housing for older people is one of the major challenges that Western countries face [3,11]. Ever increasing numbers of older people live independently in society, also referred to as ageing-in-place, which is not just related to the preferences or wishes of older people themselves [12]. Government measures, such as reforms in long-term care, also play an important role.

When taking a closer look at the Netherlands, which is an OECD member state, there are several challenges related to housing for older people. For example, in recent years many residential care homes, which are an intermediary form of housing in bridging the gap between one’s own home and a nursing home, have been closed, and many have been converted into facilities for independent living [13]. As a result of these changes in policy, a significant group of older people are in danger of getting left out: those who do not need continuous care and support, but who are nevertheless seeking the safety and jointness of a collective form of housing for older people [14,15]. Such types of housing bridge the gap between ageing-in-place and institutional care facilities, and are also referred to as co-housing communities.

The Dutch government expects that municipalities, social housing associations and market parties will take more action in the coming years, and that various new forms of housing for older people will be built [16], p.40. In particular, the supply of intermediate forms of housing for older people with low and middle incomes is limited. However, these efforts will not be successful without the participation of older people in concept and design phases of these new housing facilities.

Although some innovative housing concepts are actually founded by active groups of Dutch older people themselves [14,15], the majority of new (collective) housing for older people is established by social entrepreneurs. Social entrepreneurs, such as social housing associations and certain small and medium enterprises, are looking for innovative solutions to social problems. A social enterprise delivers a product or service just like any other enterprise and has a revenue model. However, earning money is not the main objective, it is a means of achieving the mission. Social entrepreneurs are faced with a multitude of challenges [15], such as the large number of national directives which need to be followed, and the involvement of stakeholders in concepting and decision-making. The involvement of (potential) future residents is considered to be particularly challenging, yet important to tune the needs of market supply to those of the customers. Having older people’s voices heard during the concept and design phases of the development of new housing facilities fits the goals of the age-friendly city movement, as this pertains to the domains of buildings and housing, social participation and social inclusion. Because of the aforementioned limitations, new initiatives are launched unsatisfactorily, and the current supply of housing concepts is limited or does not match the actual needs of older people [17]. Therefore, many older people in need for intermediary forms of housing are left out of the market, particularly those of low- and middle-income groups.

The growth in the demand for independent housing concepts, which can accommodate a wide range of health and social care services when needed, is simply too high. In order to successfully establish new housing concepts, entrepreneurs need to improve the active participation of older people in the concept and design phases, for instance, by taking away the limitations experienced by older people, such as the methodology chosen for this participation and the perceived freedom to express views.

The way in which older people’s participation can be organised to arrive at innovative concepts is an important part of such efforts. General lessons can be drawn from the international literature about which factors determine whether new initiatives for ‘age-friendly environments’ are successful or not. Based on a literature review, Steels [18] concluded that the following factors are particularly important: a fruitful collaboration between different stakeholders; participation of local and national governments in financing and political support; and the involvement and social inclusion of older people. Various studies into the successful establishment of new initiatives in housing and care for frail older people showed that involving older people themselves is crucial [19], for instance though community engagement [20]. However, how do you organise such participation in an innovative, facilitating and inclusive way?

In this commentary, we present an overview of the challenges of creating collective housing for older people in The Netherlands, followed by an outline of the way social entrepreneurs try to provide solutions for the market demand. The importance of end-user participation in the concept and design phases has been outlined. In the following sections, an overview is provided of the theoretical state-of-the-art, namely ways to include older people in the concept and design phases of new housing facilities. This commentary will then consider the concepts of partnership and participation. Thereafter, an overview is provided of what older people expect from participation.

## 2. Levels of Participation

According to Dedding and Slager [21] participation is a situational and interactive process in which all stakeholders in research and/or policy are in dialogue, doing justice to the lived experiences, knowledge and competences of all actors, especially individuals whose daily life and body are at stake, in all phases of the process, aiming for improvements in quality of care and a more inclusive society. Therefore, in each specific context people have their own expectations, needs and wishes during the process. Participation of older people is not only seen in the domains of housing or healthcare, but also in educational settings and the innovation of healthcare technologies [22,23,24]. Before we look at particular methods for participation, it is important to have a look at the various levels of participation that can be distinguished.

**Figure 2:**
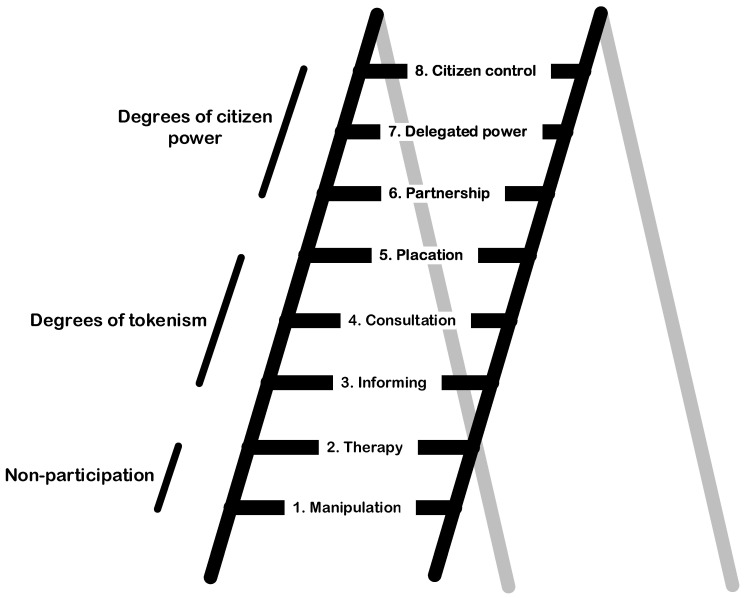
The eight rungs of the Ladder of Citizen Participation. Taken and adapted from Arnstein [25], p. 217.

The widely used participation ladder by Arnstein [25] can be taken as a starting point in shaping the various roles that older people could play (Figure 2). When looking at the roles older people could and might want to take on during the participation process, the participation ladder can be a useful tool. The rungs show the level of influence participants can have: the higher they are on the ladder, the more power the group has in determining the end product. It should be noted that it is not a goal in itself to be as high as possible on the ladder. People have different wishes and skills, and different goals might ask for a different level of participation. It is also important to note that in different stages of a project or process, different roles may be desirable.

When looking at the level of *consultation*, older people could provide information about their wishes and needs. Methods that are often used on this level of participation are surveys, interviews and focus groups [26]. Consultation often requires a relatively limited amount of time and effort of participants and could be an accessible way for different groups of sharing wishes and needs [27]. One level higher is the level of *placation*, where older people could also be asked to give advice. This would often call for a more active role of the participant, and often requires more time and skills depending on the situation and method [27]. However, the formed advice does not have to be followed and the power still lies with the other stakeholders [25]. Both consultation and placation pose a risk of ”tokenism”, where researchers or stakeholders want to—or say they—give older people a voice, but where there is no place for their actual wishes and needs or they are just being overruled [21]. In meaningful participation all perspectives influence the decision-making process [21].

On the higher rungs of the ladder, the level of power increases. In *partnership* older people would have equal power, and they can negotiate or team-up with other stakeholders. This can take different forms. Older people could become project members from an early phase on. On the highest rungs of the ladder (*delegated power* and *citizen control*) older people could have more influence in the decision-making than other stakeholders. Within the methods that are often used today, these higher levels of the ladder often require specific skills of participants, which could exclude certain groups or people and could raise questions about representation [27].

The Handbook for Participation for Older People in Care and Welfare Projects, which has been drawn up from the *Dutch National Program for Elderly Care* [28], distinguishes comparable roles (from ”listener” and ”adviser” to ”client”). Since the participation ladder is still part of the state-of-the-art in the international literature, such wording is in line with the classical terminology. There is no ideal form of participation that is suitable for all situations; it depends on the goals, wishes and skills of those involved what level of participation is appropriate. The use of the participation ladder is relevant and important because of the aforementioned bias for existing structures; it prevents certain forms of participation from being overlooked in advance.

Research into citizen participation showed that the intention to involve citizens (for example residents, clients or patients) is not a guarantee for success: (1) there is often only a limited number of people who want to actively participate; (2) the people who want to be active do not always have the skills required to do so within the methods used; and (3) people who do want to participate and have the required skills are not by definition representative of those they claim to represent [29].

## 3. Examples of Participation in the Concept and Design Phases

The design of housing for older people is a complex and dynamic process, which involves a large number of stakeholders, of whom some have specific health-related needs. The design of a building itself is characterised by dimensional, technological and stakeholder complexities that are derived from technology philosophy [30,31]. Designing homes, buildings, and neighborhoods with older adults, through exercises in participatory or co-design, could help ensure that environments are better able to facilitate healthy ageing [32]. Brookfield et al. [32] provided a critical overview of eight “less traditional” engagement techniques—walking interviews, photovoice, photo-elicitation, Talking Mats^®^, participatory mapping, drawing, model-making, and the “Design Fair”. In practice, different levels of participation can be witnessed. The levels of participation range from non-participation at the bottom, to degrees of tokenism, i.e., the passive inclusion of people, to a degree of citizen power in which people are truly participating (partnership, delegated power and citizen control).

In traditional real estate development, older people were not consulted or involved in the concept and design phases. A real estate developer or a social housing association commissioned a design from an architect, and end-users were not involved. Their needs were considered through the implementation of standards and building codes, as well as the consultation of anthropometric data. Such projects were an example of non-participation. In recent times, there has been a shift towards informing and consultation, for which panels of end-users were invited to comment on the programme of a building and designs variants. Some entities have gone beyond these stages of tokenistic participation, and have moved towards a certain degree of citizen power.

**Figure 3:**
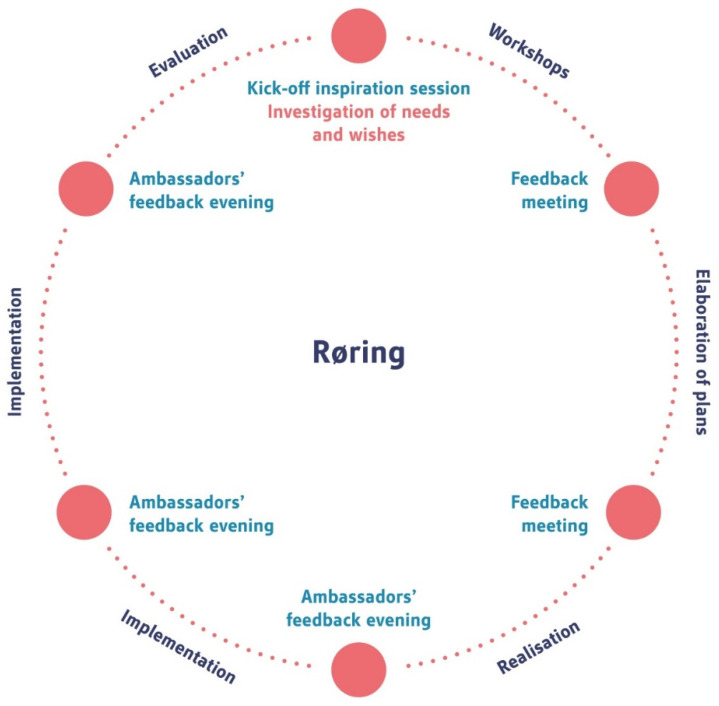
The cycle of the Røring methodology [13].

One Dutch social housing association that is specialised in housing for older people transforms vacant residential care homes into community buildings for independent living. Such transformation processes are rather iterative, and therefore, the experiences of the previous projects were turned into a methodology coined Røring [13,33]. The innovation focusses on the process of change emerging from the cocreation of participants and aiming at achieving goals in new ways [34]. Røring is a sequential methodology which involves a kick-off meeting to facilitate and inspire participants, followed by workshops leading to data analyses, translating to a greater understanding of the needs and requirements which in turn will be integrated in the implementation and realisation phase, followed by a formal evaluation [13]. Throughout each phase, feedback is required from residents in a bid to stimulate the ‘life and soul’ of the process (Figure 3). The methodology revolves around a positive and shared working goal across all interested stakeholders. This compares to rung 6 of the participation ladder (partnership). Partnership is redistributed through negotiation between citizens and power holders. Planning and decision-making responsibilities are shared, for instance, through joint committees. The Røring method is one example of a way to establish co-creation between tenants, residents and their families, the local community, long-term care organisations, municipalities and the housing association; increasing active stakeholder participation. In practice, the added value of the method and the quality of its outcomes are also dictated by the willingness of stakeholders to participate, and the level of participation, i.e., the amount of useful data shared with the social housing association and its commitment to use this input to improve the final design. The choice for the Røring method does not automatically mean that it leads to a partnership with end-users. If an organisation is not willing to embrace the outcomes of the cycle and merely sees the input as a type of free advice, a lower position on the ladder will be achieved.

Another example from the Netherlands of end-user involvement in terms of age-friendly cities, is a study by von Faber et al. [35]. This example does not relate to housing alone, but to the living environment in its entirety. The method of participatory video design was used as an empowering approach to collect experiences and perceptions of older people focusing on the age friendliness of their city or neighbourhood. The methodology provided insights in the needs and wishes of older people about the improvement and preservation of their environment. This project compares to rung 4 of the participation ladder (consultation) and rung 5 (placation), although a higher rung was aimed for. The ambition for the level of participation was higher, however the authors found that there were barriers on the organisational level, such as local policies aimed at the involvement and participation of citizen within a community. An important prerequisite for the participation in decision-making processes is to involve older people together with other stakeholders from the start. Moreover, it is fundamental that the participants jointly decide on how the results will be implemented and/or the follow-up is organised.

## 4. What Do Older People Expect from Participation?

People have different ideas and preferences regarding participation [21,36]. Groot and Abma [37] found that different generations of older people have different preferences and needs in the participation process, linked to that particular generation. When conducting an age-friendly city project in Amsterdam, Groot and Abma [37] found that people of the Baby Boom Generation (born 1940–1955) were eager to work together and participate as co-researchers. They seemed driven by action, they were motivated by creating social change, and ownership seemed important to them. A conflict emerged when other stakeholders involved were taking credit for the work of the participants. This was resolved by re-establishing ownership. In this project, participants of the Baby Boom generation formed a link to more vulnerable groups in the neighbourhood.

Another example is an action research project by Baur and Abma [38]. A group of nursing home residents (82–92 years old) was brought together in order to improve their living conditions. Participants seemed a bit shy and cautious to speak at first, and building trust was important. At first, they seemed to play down their complaints, but over time, they felt freer to speak when they found out that other residents felt the same. The sociality of the process was important to them. The nursing home residents formed a partnership and researchers facilitated empowerment.

In participation some older people might be hindered by physical or mental limitations, or they might experience a feeling of not wanting to complain due to their personal and cultural background. However, this does not mean they do not have needs or ideas that they want to be taken seriously [29,36,37,38,39]. Moreover, this does not mean they cannot or do not want to participate in more active ways as the example of Baur and Abma [38] showed. In their study, an active form of participation was facilitated and the group’s empowerment was enhanced. The employment of creative methods and creating a more responsive environment could be key in establishing meaningful participation [21].

## 5. Factors That Impact the Participation of Older People

It is valuable for all parties to talk in an early stage about the roles they will take in the participation process. Parties involved often have different ideas about goals, tasks and responsibilities, which may lead to misunderstandings, disappointments or conflicts [39]. To improve ethical participation, it is important to consider: (1) establishing a shared vision about the roles and the goals of participation; (2) the process and method; and (3) the practical aspects [39].

De Weger et al. [20] reviewed the barriers and enabling factors for engaging communities in the planning, designing, governing and delivering health and care services. They identified eight action-oriented guiding principles:

- Ensure staff provide supportive and facilitative leadership to citizens based on transparency;
- Foster a safe and trusting environment enabling citizens to provide input;
- Ensure citizens’ early involvement;
- Share decision-making and governance control with citizens;
- Acknowledge and address citizens’ experiences of power imbalances between citizens and professionals;
- Invest in citizens who feel they lack the skills and confidence to engage;
- Create quick and tangible wins;
- Take into account both citizens’ and organisations’ motivations.

Several prerequisites and success factors have been identified for an equal and constructive cooperation between older people and professionals [40,41]:

- Recognition of the participation of older people, right from the start, as a prerequisite;
- Clear agreements about objectives, tasks, responsibilities and decision-making powers and evaluation of these agreements over time;
- Reservation of time and necessary resources to shape participation;
- Taking account of specific needs, ranging from the availability and accessibility of information to physical limitations or organisational capacity;
- Provision of necessary organisational, substantive and strategic support.

Furthermore, the equal and constructive cooperation between older people and professionals also requires an attitude that understands participation as a two-way street, in which plans and ideas can be discussed and adjusted from both sides, and that recognises and appreciates differences of opinion, including the differences among older people themselves. Having an attitude of openness towards the initiatives of older people themselves, their knowledge and experiences is important, in which professionals and older people see each other as equal partners with different backgrounds and experiences, insights and expertise.

Regular feedback should be provided to participants about what has been done with the input of those involved, and how this has influenced the decision-making or the further design of a project. In the same way regular contacts should be maintained with the wider target group and results should not only be disseminated to those directly concerned, but also communicated to the wider community [40,41].

With regard to the participation of older people of an ethnic minority, a number of additional points of attention can be formulated. The involvement of older people should take place at the earliest possible stage, at the very first moments of conceptualisation, in order to make them co-owners of the project or initiative in question [42]. Taking the participation of older immigrants seriously, requires participation to be shaped in a way that does justice to their life world and life experience. Too often, projects are set up in a way that takes little account of the possibility that older immigrants may need a more culturally sensitive form of contact. It requires a pro-active attitude and active engagement, meeting people in their own environment, using the communication channels and forms of communication that are common in the various communities. An additional problem may be that the number of (potential) volunteers among older immigrants known to organisations is often still relatively small, with the risk that often it is the same people who are invited to participate and who, as a consequence, may therefore be over-asked. It also may lead to a one-sided view of the target group. It is important to recognise the diversity amongst older people with a migration background, and prevent one and the same person from being asked to speak on behalf of the very diverse group of older immigrants [42].

Finally, Machielse et al. [43] described seven conditions for a ‘vibrant residential community’ that promote the self-organising capacity and active involvement of residents and enable them to develop their own initiatives:

- Commitment of the organisation(s) involved to the objectives of the initiative and the willingness to cooperate with residents and to facilitate them;
- A clear picture of the existing situation; the living environment, the structures, the needs and preferences of residents;
- Formulation of clear and realistic goals, based on a clear understanding of the current situation;
- The presence of a group of motivated residents, who are open to new ideas, willing to offer space to other residents and, if necessary, to support them; who are able to set activities in motion and to attune them to the needs and pace of other residents and, by doing so, gain support for the initiative;
- Clear communication to residents, creating clarity about the background, objectives, tasks and responsibilities;
- The availability of an open and accessible common space where residents can meet;
- The support of a facilitating professional, aimed at activating and supporting the residents’ capacity for self-organisation.

The abovementioned recommendations can help steer the process of participation of older people in the concept and design phases of new forms of co-housing, and help select the most fitting rung on the ladder of participation. Older people should be given the choice to decide (in dialogue with initiators) on the extent to which they wish to participate in the concept and design phases.

## 6. Afterthoughts and Recommendations

This commentary describes different levels of participation of older people in the design process of new housing concepts. It should, however, also be acknowledged that ageing-in-place goes beyond the creation of appropriate housing. A healthy physical and social living environment is just as important, as lies also at the core of the age-friendly agenda. Up until now, spatial planning processes often take place without active participation of older people. As a result, the needs and preferences of older people are often insufficiently addressed in spatial planning [44]. Moreover, in those cases where older people are being consulted, this is mostly limited to relatively healthy older people. More frail older people, for instance those who are chronically ill, have a low socio-economic status or are from an ethnic minority group, participate even less in spatial planning processes [45]. It is, therefore, recommended that, in order to facilitate ageing in place, participation of older people should be realised in both design processes of new housing concepts as in the direct living environment.

The first steps in this direction are currently being undertaken by the Dutch National Institute for Public Health and the Environment in the project “*Working together on healthy design principles with a special focus on (frail) older people*” [46]. An important aim of this project is to identify the methods that are currently being used for the participation of older people in spatial planning and the experiences of different actors (including older people, community policy officers, researchers) with these methods. The outcomes of this inventory will be incorporated in a roadmap for healthy design for older people, including the relevant steps for healthy design, healthy design principles and the related evidence-base.

When designing social living environments for older people or age-friendly cities, van Hoof et al. [47] postulated that such attempts may introduce features—intended at improving the quality of life of older people—that may actually be based on age-stereotypes, both positive and negative ones. In practice, the phenomenon of ageism may interact with the age-friendly developments, which may sound counterintuitive as the process of a city towards becoming age-friendly is often perceived as something positive. Ageism as a concept was coined by Butler [48], who referred to it as prejudice on the basis of age. In contrast, the recognition of the mere existence of implicit and explicit ageism in the built environment and its potential impact on the design of age-friendly cities are understudied and unexplored domains, thereby urgently raising the importance for stakeholders to address the concept. One example of explicit ageism in the urban environment is poor or absent accessibility for older people, who, are often completely ignored by architects, designers and urban planners [47]. A recent study from the United Kingdom suggest people may delay having adaptations, because of perceived stigmatising associations with decline and vulnerability [49]. This is why it is so relevant to actively include older people in the decision-making processes of new housing and, also, urban planning concepts.

The participation of older people in decision making concerning new housing concepts can take place through the various rungs of the participation ladder. The higher up the ladder, the more participants can be involved and their voices be heard. The success of the innovation rests on the level of participation and involvement of a wide variety of interests—older people in the various rungs of the participation ladder—but also the beneficiaries of the innovation as well as the producers and suppliers [50]. The commentary has shown that the intention to involve people is not a guarantee for success and that the recruitment of active participants is limited by a lack of volunteers or impacted by the skills of potential participants. Some older people with dementia may have great challenges to be included in any kind of participation process, although stimulating and engaging methods for their participation do exist [51]. In addition, there may be a bias in the representation; are the participants representatives of the group of people they wish to represent? This requires teams engaging in participation projects to actively address these potential shortfalls in order to make the most out of a participation project. The methods chosen for the participation of people can differ for each and every project, depending on the scale of the project, the type of the housing that needs to be addressed, the number of participants, and the time a design and realisation cycle takes. Their appropriateness and actual contribution to the quality of the final design should be studied in the future. This is particularly important as the Dutch government wants to combine and simplify the regulations for spatial projects through the new Environment and Planning Act, which calls for the active participation of all citizens in spatial projects.
